# Nutrient availability shapes the microbial community structure in sugarcane bagasse compost-derived consortia

**DOI:** 10.1038/srep38781

**Published:** 2016-12-12

**Authors:** Bruno L. Mello, Anna M. Alessi, Simon McQueen-Mason, Neil C. Bruce, Igor Polikarpov

**Affiliations:** 1 Instituto de Física de São Carlos, Universidade de São Paulo, Av. Trabalhador São-carlense 400, São Carlos, SP, 13560-970, Brazil; 2 Department of Biology, University of York, Wentworth Way, York, YO10 5DD, UK

## Abstract

Microbial communities (MCs) create complex metabolic networks in natural habitats and respond to environmental changes by shifts in the community structure. Although members of MCs are often not amenable for cultivation in pure culture, it is possible to obtain a greater diversity of species in the laboratory setting when microorganisms are grown as mixed cultures. In order to mimic the environmental conditions, an appropriate growth medium must be applied. Here, we examined the hypothesis that a greater diversity of microorganisms can be sustained under nutrient-limited conditions. Using a 16 S rRNA amplicon metagenomic approach, we explored the structure of a compost-derived MC. During a five-week time course the MC grown in minimal medium with sugarcane bagasse (SCB) as a sole carbon source showed greater diversity and enrichment in lignocellulose-degrading microorganisms. In contrast, a MC grown in nutrient rich medium with addition of SCB had a lower microbial diversity and limited number of lignocellulolytic species. Our approach provides evidence that factors such as nutrient availability has a significant selective pressure on the biodiversity of microorganisms in MCs. Consequently, nutrient-limited medium may displace bacterial generalist species, leading to an enriched source for mining novel enzymes for biotechnology applications.

Recent interest in development of renewable energy such as biofuels has led to multiple reports exploring natural ecosystems for novel enzymes involved in the degradation of lignocellulose^1^,^2^,^3^,^4^,^5^,^6^. The enzymatic conversion of plant residues represents a major cost of biofuel production, limiting its economical competitiveness and sustainability. The isolation of lignocellulose degrading microorganisms has been reported from environments such as compost^7^,^8^,^9^,^10^,^11^, soil^11^,^12^,^13^, water^13^, animal waste slurry^11^, and invertebrates^14^,^15^. Metagenomics approaches and the rapid development of new sequencing techniques have greatly enhanced our understanding on how these complex microbial communities (MCs) are structured and how they respond to environmental factors. For instance, amplicon based sequencing of the ribosomal RNA coding regions and other molecular markers nowadays provides quick and affordable metadata on the composition of a MC. Since microorganisms represent the majority of the biodiversity found in terrestrial ecosystems and are intimately involved in ecosystem functions^16^, metagenomics is one of the key technologies used to access and investigate this genetic reservoir^17^. Studies on environmental MCs using -omics technologies, therefore, offer opportunities for improving biotechnological processes involved in wastewater treatment, soil remediation, agroindustrial waste degradation and second-generation biofuels production^18^,^19^,^20^.

Since most of metagenomics studies rely on collecting sample directly from the environment, the resulting data often shows a low number of target genes. In order to improve detection rate of desired properties, enrichment techniques are often applied where the environmental sample is inoculated into a liquid medium with a supplement that will force the enrichment e.g. plant biomass, xenobiotics etc. By using this approach, recent metagenome studies on rumen, soil or compost derived microbial consortia showed successful enrichment of enzymes involved in lignocellulose degradation^21^,^22^. The resulting approach will determine predominance of different specialized taxa in the enriched community based on availability of, demand for and consumption rate of nutrients in the applied growth medium^23^. Hence, the resource availability in those pre-set cultures will affect the complexity, behavior and competitive ability of microorganisms as shown initially by Tilman^24^. It is also known that a more diverse resource will sustain greater biodiversity in MCs by reducing interspecific competition due to better distribution of resources between species^25^. There is a growing interest to understand how complex MCs such as those present in human and animal guts, soil or oceans respond to environmental factors such as nutrient availability.

In the current study, exposing a compost-derived microbial consortium to nutrient limited minimal medium (MM) and nutrient rich medium was investigated. We determined how the composition of a MC and lignocellulolytic activity are affected by resource availability using amplicon based metagenomics and selective plate screening. A nutrient rich medium supplemented with lignocellulosic biomass provides multiple energy sources and should allow more generalist microorganisms to thrive. In contrast, a MM, with lignocellulosic biomass and no additional carbon sources, should enhance growth of specialized lignocellulolytic degraders creating enriched MCs. Our study demonstrates that microbial consortia obtained from MM have in fact greater diversity and proliferation of lignocellulose-degrading microorganisms as compared with MC grown in nutrient rich medium.

## Results

### Screening for cellulolytic microorganisms in compost-derived microbial consortia

**Figure 1:**
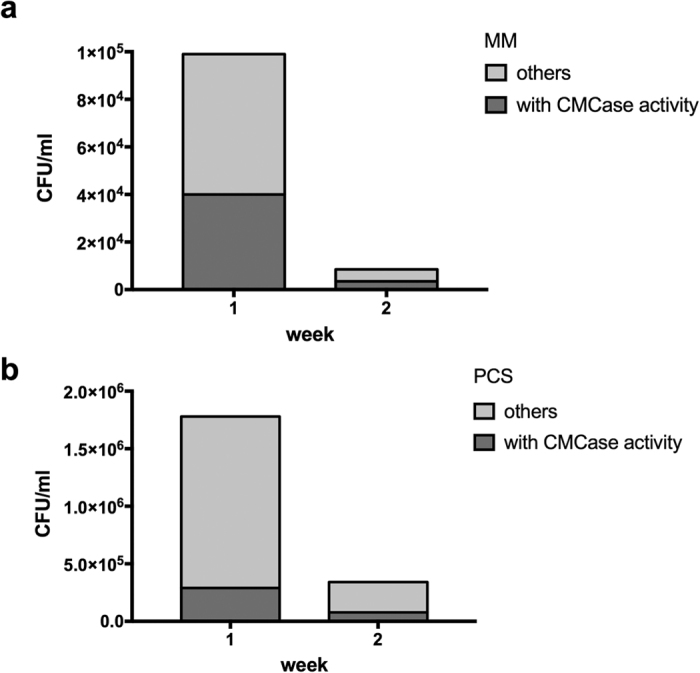
Number of colony forming units (CFUs) of compost-derived consortia grown in (**a**) minimal medium and (**b**) PCS. Supernatant from liquid cultures after incubation for one and two weeks was collected and spread as serial dilutions on solid agar plates supplemented with carboxymethyl cellulose (CMC). Subsequently, colonies were removed and plates were stained with Congo red. The proportion of isolates producing clearance zones − indicative to cellulolytic activity − is presented in the figure.

In order to estimate the number of culturable microbial cells in the *in vitro* composting cultures, we used agar plates inoculated with a serial dilution of the MC. After one week, the PCS-grown cultures had 18 times higher cell density than the MM-grown cultures (Fig. 1). Two-week old cultures showed dramatic decrease in the cellular density compared to week 1, by having 10- and 5-fold lower number of CFU/ml in the MM and PCS cultures, respectively. In contrast, the proportion of microorganisms with cellulase activity measured by the number of colonies displaying clearance zones on carboxymethyl cellulose (CMC) plates was significantly smaller in PCS culture than for those obtained from MM. This observation was confirmed by our qualitative sugarcane biomass assessment. The sugarcane bagasse that remained after microbial growth was filtered, dried and visually examined for its breakdown. Biomass obtained from MM-grown cultures (Supplementary Fig. 1) showed a reduction in particle size and biomass darkening. The PCS-grown cultures, which offered more accessible carbon sources than sugarcane bagasse, showed reduced lignocellulose breakdown even after 5-weeks incubation. Size and morphology of sugarcane remained unchanged in those cultures, mainly due to low abundance of specialized lignocellulolytic microorganisms that was observed during CMC-plate assay.

### Evaluation of microbial diversity in compost-derived consortia using high-throughput sequencing

A total of 1,224,357 (n = 8, 170,000 reads per samples) 16 S rRNA sequence reads were obtained for the *in vitro* composting MCs. The minimum number of reads mapped to a sample was 46,999 with the exception of sample PCS-5, for which 11,603 reads were obtained. The number of sequenced reads for individual samples and assigned OTUs are given in Table 1.

**Figure 2:**
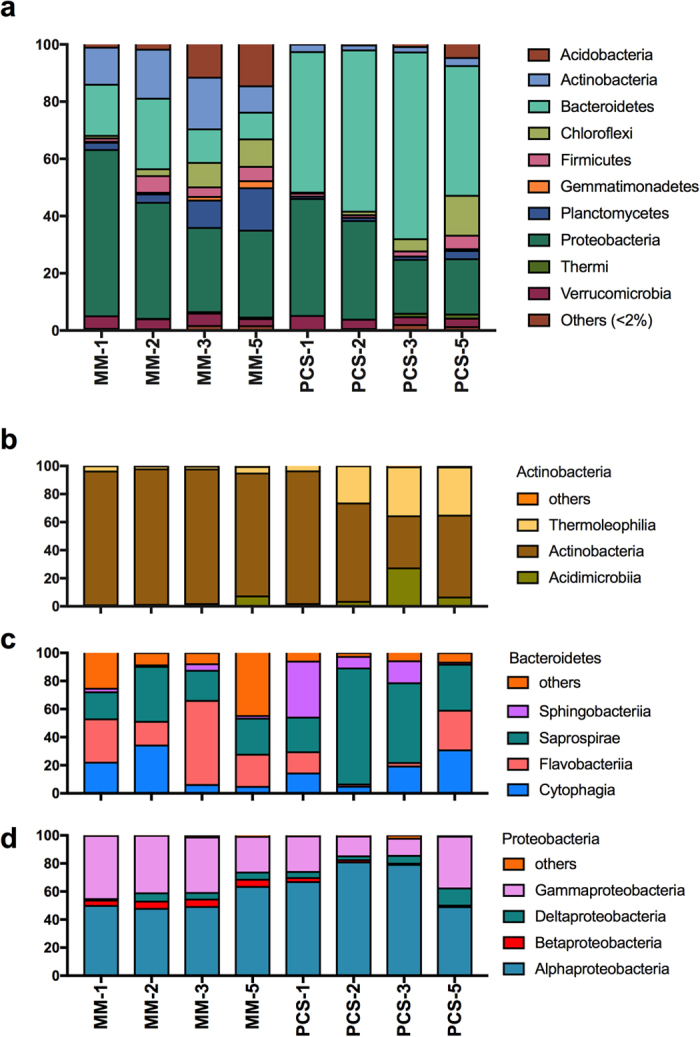
Relative abundance of bacterial (**a**) phyla and (**b**) classes for phylotypes identified in compost-derived consortia grown on minimal medium and PCS using 16 S rRNA sequencing. Number next to the medium abbreviation represents week when sample was collected. y-axis = relative abundance [%].

Taxonomic distribution of our *in vitro* composting liquid cultures was examined at the level of phylum (Fig. 2a) and class (Fig. 2b–d). Relative abundance at the phylum level varied depending on the growth medium used. The compost-derived consortium grown on MM was dominated by Proteobacteria (58%), Bacteroidetes (18%) and Actinobacteria (13%), accounting for nearly 90% of the total diversity in week 1. The OTUs assigned to Bacteroidetes and Actinobacteria showed gradual increase to 25% and 18%, respectively, during mid time points, followed by a decrease in the week 5 sample. Further, phylotypes assigned to lineages such as Acidobacteria, Chloroflexi, Firmicutes and Planctomycetes initially accounted for 6% of all picked OTUs. Those phyla showed a gradual increase in week 5 cultures, proving that the diversity of bacterial community at phylum level, increased over time. The phylum Actinobacteria was strongly dominated by the class Actinobacteria with relative abundance higher than 87% over the 5-week time course. In contrast, phylum Bacteroidetes showed more variation over time, and was dominated by the representatives of class Cytophagia, Flavobacteria and Saprospirae. Further, members of Alphaproteobacteria and Gammaproteobacteria were recovered in all the samples with a relative abundance higher than 88% of all Proteobacteria during the tested time points.

The MC grown on PCS medium was dominated by OTUs belonging to Bacteroidetes (49%) and Proteobacteria (41%) accounting for 90% abundance in the week 1 culture. The phylum Bacteroidetes accounted for more than 44% abundance at all time points taken, showing a clear dominance in the cultures. Other two phyla that showed an increase in the relative abundance were Chloroflexi (from 0.3% to 14%) and Firmicutes (from 1% to 5%). Within Bacteroidetes phylum, members of Saprospirae dominated PCS cultures at the class level, however Cytophagia and Flavobacteria showed increasing abundance over time. Proteobacteria were strongly dominated by Alphaproteobacteria and Gammaproteobacteria similarly to MM cultures.

**Figure 3:**
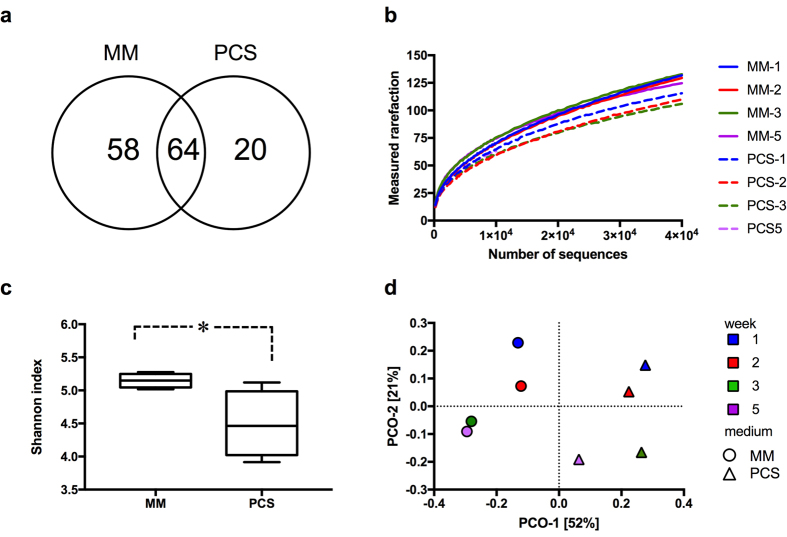
Diversity analysis of compost-derived consortia. (**a**) Venn diagram presenting number of unique and shared OTUs for MM- and PCS-grown compost derived consortia. (**b**) Rarefaction curves showing the calculated rarefaction using phylogenetic diversity (PD) versus the number of reads sequenced per sample. Other diversity indices, such as chao1 and observed_species, were also tested showing similar results. (**c**) Box-whisker plot of the calculated Shannon’s diversity index. The results were grouped by the medium used for growth. The asterisk (*) denotes a significant difference (p ≤ 0.05, Student’s t-test) between MM- and PCS-grown cultures diversity indexes. (**d**) PCoA of weighted UniFrac distances. Consortia obtained from the same growth medium clustered together, as presented in the figure.

To further analyze which phyla were primarily responsible for the differences between the MM and PCS grown communities, we applied a SIMPER test (Table 2). The OTUs classified to genus level were grouped to phylum and class level after analysis. The phyla Bacteroidetes (classes Saprospirae and Sphingobacteria), Proteobacteria (classes Gammaproteobacteria and Alphaproteobacteria) and Actinobacteria (class Actinobacteria) were responsible for nearly 60% of the observed dissimilarity between the MM and PCS consortia based on Bray-Curtis distance. Those linages were the most abundant in our cultures and accounted for 37% to 83% relative abundance depending on the sample. The major contributors to community differences were also analyzed using SIMPER test at genus level. Amongst the twenty most abundant OTUs in each medium (Supplementary Table 1), five phylotypes were present in both consortia. In both MM and PCS cultures, 70% of OTUs could not be classified at genus level, indicating presence of microorganisms belonging to previously non-described genera. The twenty most abundant OTUs accounted for 57% and 75% of all assigned OTUs in MM and PCS cultures, respectively. This result shows that few species dominated the cultures and reveals that the microbial diversity in PCS-grown culture was lower than in MM consortia. The higher complexity of MM cultures was confirmed by a presence of more unique OTUs accounting for 41% of total identified phylotypes (Fig. 3a). In addition, SIMPER analysis showed that 17 OTUs observed in MM cultures accounted for 50% of the total sequenced reads. When PCS consortia were investigated, 8 OTUs represented the same number of reads (Supplementary Table 1).

### Diversity analysis

Alpha diversity rarefaction curves (Fig. 3b) show a small decrease in microbial species richness with incubation time. Even with 40,000 sequences per sample, the curves did not reach an asymptote, indicating a high microbial diversity in the samples and that a larger sequencing effort would need to be employed to identify all the microbes present. The Shannon index (Fig. 3c) confirmed those observations with a greater microbial diversity in MM- than PCS-grown cultures.

Principal coordinate analysis (PCoA) using weighted UniFrac distances (Fig. 3d) was used to estimate the phylogenetic differences between samples. PCoA showed that the consortia derived from MM and PCS medium were classified into two distinct clusters. The principal coordinate 1 (PCO-1) was sufficient for total separation of those two groups based on the growth medium used for culturing. Within each cluster, the PCO-2 allowed the samples to be grouped based on their growth time showing demarcation of early and later time points.

## Discussion

Bacteria that can grow in pure culture under laboratory conditions represent a small fraction of the total microbial diversity that can be found in nature. Studies have estimated that only up to 1% of the soil microorganisms could proliferate in standard growth media^26^,^27^,^28^. Many bacteria have specific nutrient or chemical requirements, naturally present in their environment^29^,^30^ but difficult to replicate in the laboratory. In addition, syntrophic relationships in complex MCs are a limiting factor in culturing many microbes. However, this uncultured part of the MC is capable of synthesizing novel unknown natural products and plays a critical role in carbon, nitrogen and nutrient recycling^31^ such as lignocellulose breakdown.

Microbial consortia have been demonstrated to enhance lignocellulose degradation compared to monocultures^32^,^33^,^34^,^35^ that often display lower biodegradation rates^36^. Previous studies on employing microbial consortia for lignocellulose breakdown utilized both complex^37^,^38^,^39^ and defined media^40^,^41^,^42^.

In the present work, we evaluated the changes in compost-derived MCs cultured in a presence of nutritionally rich and minimal growth medium. A complex PCS medium provided a rich set of nutrients from yeast extract and peptone such as vitamins and amino acids to sustain microbial growth to a high density. In contrast, lignocellulose degradation was suppressed due to availability of other more accessible energy sources. The proportion of microorganisms degrading lignocellulose as assessed by CMC-screening was low and visual inspection of SCB biomass lacked morphological changes at the end of experiment. We expected to induce lignocellulolytic MC using a defined MM supplemented with sugarcane bagasse as a sole carbon source. This medium contains basic components for bacterial growth such as ions, electrolytes (provided as trace elements mixture) and nitrogen, but no carbon source. The MM grown consortia displayed a higher proportion of cellulolytic microorganisms than the PCS cultures and efficiently degraded SCB over five-week trial as observed in our qualitative assessment of weekly-collected biomass. Quantitative biomass analysis that relies on saccharification using hydrolysis with acid, followed sugar separation by a high performance anion exchange chromatography has been previously used to evaluate compositional changes in lignocellulose residues^43^,^44^. However, in our study we were unable to remove all the microbial biomass from the plant residues, these cells also underwent hydrolysis during the acid treatment releasing amino sugars from the bacterial cell wall and neutral sugars from the extracellular bacterial matrix. These microbially derived sugars affected the accuracy of the HPLC method showing inconsistent results.

To evaluate the complexity of our composting communities, we applied amplicon sequencing of V4 region of 16 S rRNA gene using methods and standards recommended by the Earth Microbiome Project^45^. Amplicon sequencing of 16 S rRNA is widely used approach to estimate the relative abundance of microorganisms in the MCs. However, since the method is based on PCR amplification, multiple factors have been reported to affect its performance including the DNA extraction procedure, primer design, DNA polymerase and number of amplification steps^46^. Based on alpha diversity measurements we found that bacterial diversity and richness was significantly higher in MM grown consortia. As reported by Mitri *et al*.^47^, nutrient limitation is a key mechanism underlying the maintenance of diversity in cell groups. This observation is in agreement with our study. The number of cells in the nutrient rich medium was higher than in MM-grown cultures leading to expansion of fast diving cells, which promoted the loss of culture’s diversity. Based on our results, we conclude that MM is more suitable medium to study MCs in the laboratory setting because it mimics natural conditions where cell densities and growth rates can be much lower than using nutrient rich medium.

The phylogenetic distances using UniFrac showed separation of MM and PCS grown cultures and clustering of early and late time points. Both consortia showed presence of phylotypes from the phylum Proteobacteria whose members are known for metabolic diversity^48^. Members of Bacteroidetes and Actinobacteria present in both communities have been linked to degradation of complex polymers in compost^1^,^6^ and have been reported in composting piles previously^49^,^50^. Interestingly, MM community was enriched in phylotypes including known and characterized genera such as *Cellulomonas, Paenibacillus* and *Cellvibrio*. Isolates from latter genera were shown to be involved in degradation of various plant materials^49^,^51^,^52^. MM grown consortia also showed high relative abundance of unknown OTUs from diverse families. PCS most abundant OTUs were species that assimilate simple carbon sources such as *Sphingobacterium* and *Asticcacaulis. Shinogobacterium* sp. was reported as prevalent producer of glycosyl hydrolases from the CAZy families 2 and 3 in metagenomic studies of soil-derived microbial consortia and major contributor to deconstructing the hemicellulosic part of plant cell wall in soil^21^. In our PCS cultures the degradation of SCB was limited based on the visual evaluation of the remaining biomass. High relative abundance of *Sphingobacterium* sp. indicates the presence of short oligosaccharides in the medium, probably derived from the components of yeast extract.

In conclusion, our work tested the hypothesis that nutrient resources in the growth medium used in laboratory-based experiments, shapes MCs structure. Using amplicon sequencing of 16 S rRNA genes, we showed significant differences in dominant microbial taxa of compost-derived consortia grown in a nutrient limited and rich conditions. We conclude that a number of microbes are required to effectively break down recalcitrant substrates such as sugarcane bagasse, leading to a selective enrichment of specialized group of microorganisms. Consequently, nutrient-limited media can be used to attend diverse biotechnological needs when displacement of bacterial generalist species and enrichment of specific MCs is desired.

## Materials and Methods

### Sample collection and culture

Composting samples were collected between January and April 2013 from the São Paulo University Recycling Project (São Carlos campus). The samples were collected during the final mesophilic phase at locations 30 cm below the surface. A homogenized composting sample (250 mg) was used to inoculate 25 mL of either complex PCS (0.1% yeast extract, 0.5% peptone, 0.5% CaCO_3_, 0.5% NaCl pH 8.0)^39^ or MM (KCl 0.52 g/L, KH_2_PO_4_ 0.815 g/L, K_2_HPO_4_ 1.045 g/L, MgSO_4_ 1.35 g/L, NaNO_3_ 1.75 g/L, Hutner’s trace elements^53^). Cultures were supplemented with 2% (w/v) sugarcane bagasse and incubated at 30 °C with 150 rpm agitation for five weeks. Sugarcane bagasse was kindly provided by the Cosan Group (Ibaté, São Paulo, Brazil). Prior to use, it was washed with hot water (50 °C) and dried in a 50 °C oven.

### Cellulase screening on solid media

A ten-fold serial dilution of the composting cultures was spread on agar plates containing the PCS or MM supplemented with 1% (w/v) carboxymethyl cellulose (CMC). MM was also supplemented with 1% (w/v) dextrose to facilitate growth of microorganisms unable to degrade CMC. Plates were incubated at 30 °C for one week and the number of colony-forming units (CFUs) was counted. Subsequently, colonies were removed from plates with water followed by staining with 1% (w/v) Congo red dye for 15 min and destaining with 1 M NaCl for 20 min. The cellulolytic microorganisms that secrete endocellulases cause cleavage of the CMC backbone resulting in formation of a clearance zone around colonies upon staining with Congo red.

### 16 S rRNA gene sequencing

The whole microbial culture was centrifuged at 2,000 × g for 10 min and the pellet was used for DNA extraction. DNA was extracted from 50 mg pellet using the FastDNA Spin Kit for Soil (MP Biomedicals, Solon, OH, USA) according to the supplier’s recommendations. The V4 hypervariable region of the bacterial 16 S rRNA gene was amplified using 50 ng of metagenomic DNA as template and modified universal bacterial primers 515 F (5′-TCGTCGGCAGCGTCAGATGTGTATAAGAGACAGGTGCCAGCMGCCGCGGTAA-3′) and 806 R (5′-GTCTCGTGGGCTCGGAGATGTGTATAAGAGACAGGGACTACHVGGGTWTCTAAT-3′), with Illumina adapter overhang sequences indicated by underline. Amplicons were obtained by PCR using the following condition: initial denaturation at 94 °C for 2 min, followed by 25 cycles of 94 °C for 30 sec, 57 °C for 60 sec, 72 °C for 40 sec, and a final elongation step at 72 °C for 10 min. The fragments were cleaned using the Wizard SV gel and PCR Clean-Up System (Promega, Madison, WI, USA). Samples were indexed with the Nextera DNA Library Prep Kit (Illumina, Carlsbad, CA, USA). DNA concentration was measured with the KAPA Library Quantification Kit (Kapa Byosystems, Wilmington, MA, USA) according to the manufacture’s recommendations. Samples were pooled in equimolar amounts and sequenced on a MiSeq (Illumina, Carlsbad, CA, USA) producing 2 × 150 bp paired-end reads.

### Bioinformatics and data analysis

The paired-end reads were merged using Fast Length Adjustment of SHort reads (FLASH, v1.2.7)^54^. The Quantitative Insights Into Microbial Ecology (QIIME, v1.8.0)^55^ was used to perform data analysis. Reads were quality filtered (at Phred score > Q20) and operational taxonomic units (OTUs) were picked at 97% identity using the UCLUST algorithm^56^. The UCHIME algorithm^57^ was used to detect and filter chimeras. OTU was assigned to taxonomic groups using the GreenGenes database^58^ released on May 2013 and RDP Classifier. Singleton reads were filtered to reduce noise and variability. QIIME was also used to calculate the alpha and beta diversity after data sets were rarefied to 40 k and 10 k reads per sample, respectively. PCoA was used to visualize clustering of samples based on their weighted UniFrac distance matrices. PAST (v3.11)^59^ was used to calculate Shannon’s diversity index (with the 10 k reads rarefied sample) and similarity percentage analysis (SIMPER) to determine which OTUs contribute more to dissimilarity between communities.

## Additional Information

**How to cite this article**: Mello, B. L. *et al*. Nutrient availability shapes the microbial community structure in sugarcane bagasse compost-derived consortia. *Sci. Rep.* **6**, 38781; doi: 10.1038/srep38781 (2016).

**Publisher's note:** Springer Nature remains neutral with regard to jurisdictional claims in published maps and institutional affiliations.

## Supplementary Material

Supplementary Information

## Figures and Tables

**Table 1:** Number of raw and filtered 16 S rRNA reads used for OTU clustering from compost-derived consortia.

Community	Number of reads	Number of reads used for OTU picking	Number of picked OTUs	Number of rarefied OTUs
MM-1	444,182	405,578	411	195
MM-2	211,159	191,892	373	194
MM-3	87,256	78,782	347	238
MM-5	55,113	50,223	322	237
PCS-1	46,999	42,216	235	169
PCS-2	290,779	272,638	303	158
PCS-3	77,266	72,558	228	155
PCS-5	11,603	10,480	183	162

**Table 2:** SIMPER analysis showing which phyla and classes of microorganisms were primarily responsible for Bray-Curtis dissimilarity between MM and PCS compost-derived communities.

Phylum	Class	Mean abundance in MM(%)	Mean abundance in PCS(%)	Contribution to dissimilarity(%)	Cumulative contribution to dissimilarity(%)
Bacteroidetes	Saprospirae	4.4	26.9	22.4	22.4
	Sphingobacteria	0.4	8.5	8.1	30.4
	Cytophagia	3.3	8.7	6.4	36.9
	Flavobacteria	4.6	5.5	4.2	41.1
	Other	2.9	2.8	3.0	44.1
Proteobacteria	γ-proteobacteria	15.1	6.0	9.4	53.5
	α-proteobacteria	19.9	19.3	7.8	61.2
	Other	3.5	2.3	2.2	63.5
Actinobacteria	Actinobacteria	13.2	1.5	11.6	75.1
	Other	0.8	0.7	0.6	75.6
Acidobacteria	—	7.4	1.6	6.5	82.2
Planctomycetes	—	7.3	1.5	5.8	88.0
Chloroflexi	—	5.2	4.8	5.2	93.2
Firmicutes	—	3.7	2.0	2.3	95.5

The cumulative contribution to dissimilarity represents the summed contribution to dissimilarity of afore presented taxa.

## References

[b1] Ryckeboer J. . A survey of bacteria and fungi occurring during composting and self-heating processes. Annals of Microbiology. 53, 349–410 (2003).

[b2] Ishii K. & Takii S. Comparison of microbial communities in four different composting processes as evaluated by denaturing gradient gel electrophoresis analysis. J. Appl. Microbiol. 95, 109–119 (2003). 12807460 10.1046/j.1365-2672.2003.01949.x

[b3] Steger K., Eklind Y., Olsson J. & Sundth I. Microbial community growth and utilization of carbon constituents during thermophilic composting at different oxygen levels. Microb. Ecol. 50, 163–171 (2005). 16184337 10.1007/s00248-004-0139-y

[b4] Takebayashi S., Narihiro T., Fujii Y. & Hiraishi A. Water availability is a critical determinant of a population shift from proteobacteria to actinobacteria during start-up operation of mesophilic fed-batch composting. Microbes and Environments. 22, 279–289 (2007).

[b5] Vargas-García M. C., Suárez-Estrella F., López M. J. & Moreno J. Microbial population dynamics and enzyme activities in composting processes with different starting materials. Waste Manag. 30, 771–778 (2010). 20096556 10.1016/j.wasman.2009.12.019

[b6] Partanen P., Hultman J., Paulin L., Auvinen P. & Romantschuk M. Bacterial diversity at different stages of the composting process. BMC Microbiol. 10, 94 (2010). 20350306 10.1186/1471-2180-10-94 PMC2907838

[b7] Amore A. . Industrial waste based compost as a source of novel cellulolytic strains and enzymes. FEMS Microbiol. Lett. 339, 93–101 (2013). 23181595 10.1111/1574-6968.12057

[b8] Sizova M. V., Izquierdo J. A., Panikov N. S. & Lynd L. R. Cellulose- and xylan-degrading thermophilic anaerobic bacteria from biocompost. Appl. Environ. Microbiol. 77, 2282–2291 (2011). 21317267 10.1128/AEM.01219-10 PMC3067422

[b9] Kang M. S. . Cellulomonas composti sp. Nov., a cellulolytic bacterium isolated from cattle farm compost. Int. J. Syst. Evol. Microbiol. 57, 1256–1260 (2007). 17551039 10.1099/ijs.0.63974-0

[b10] Fathallh Eida M., Nagaoka T., Wasaki J. & Kouno K. Isolation and characterization of cellulose-decomposing bacteria inhabiting sawdust and coffee residue composts. Microbes Environ. 27, 226–233 (2012). 22353767 10.1264/jsme2.ME11299 PMC4036048

[b11] Kim Y. K., Lee S. C., Cho Y. Y., Oh H. J. & Ko Y. H. Isolation of cellulolytic bacillus subtilis strains from agricultural environments. ISRN Microbiol 2012, 650563 (2012). 23724328 10.5402/2012/650563 PMC3658498

[b12] Elberson M. A. . Cellulomonas persica sp. Nov. And cellulomonas iranensis sp. Nov., mesophilic cellulose-degrading bacteria isolated from forest soils. Int. J. Syst. Evol. Microbiol. 50, 993–996 (2000). 10843037 10.1099/00207713-50-3-993

[b13] Heck J. X., Hertz P. F. & Ayub M. A. Z. Cellulase and xylanase production by isolated amazon bacillus strains using soybean industrial residue based solid-state cultivation. Brazilian Journal Of Microbiology. 33, 213–218 (2002).

[b14] Gupta P., Samant K. & Sahu A. Isolation of cellulose-degrading bacteria and determination of their cellulolytic potential. Int J Microbiol. 2012, 578925 (2012). 22315612 10.1155/2012/578925 PMC3270400

[b15] Kern M. . Structural characterization of a unique marine animal family 7 cellobiohydrolase suggests a mechanism of cellulase salt tolerance. Proc. Natl. Acad. Sci. USA 110, 10189–10194 (2013). 23733951 10.1073/pnas.1301502110 PMC3690837

[b16] Madsen E. L. Microorganisms and their roles in fundamental biogeochemical cycles. Curr. opin. biotechnol. 22, 456–464 (2011). 21333523 10.1016/j.copbio.2011.01.008

[b17] Duan C. J. & Feng J. X. Mining metagenomes for novel cellulase genes. Biotechnol. Lett. 32, 1765–1775 (2010). 20640872 10.1007/s10529-010-0356-z

[b18] Curtis T. P., Head I. M. & Graham D. W. Theoretical ecology for engineering biology. Environ. Sci. Technol. 37, 64a–70a (2003). 10.1021/es0323493 12630455

[b19] Rittmann B. E. . A vista for microbial ecology and environmental biotechnology. Environ. Sci. Technol. 40, 1096–1103 (2006). 16572761 10.1021/es062631k

[b20] Castillo J. M., Romero E. & Nogales R. Dynamics of microbial communities related to biochemical parameters during vermicomposting and maturation of agroindustrial lignocellulose wastes. Bioresour. Technol. 146, 345–354 (2013). 23948272 10.1016/j.biortech.2013.07.093

[b21] Jiménez D. J., Chaves-Moreno D. & Van Elsas J. D. Unveiling the metabolic potential of two soil-derived microbial consortia selected on wheat straw. Sci. Rep. 5, 13845 (2015). 26343383 10.1038/srep13845 PMC4561380

[b22] Hess M. . Metagenomic discovery of biomass-degrading genes and genomes from cow rumen. Science. 331, 463–467 (2011). 21273488 10.1126/science.1200387

[b23] Hibbing M. E., Fuqua C., Parsek M. R. & Peterson S. B. Bacterial competition: surviving and thriving in the microbial jungle. Nat. Rev. Microbiol. 8, 15–25 (2010). 19946288 10.1038/nrmicro2259 PMC2879262

[b24] Tilman D. Resource competition between plankton Algae: an experimental and theoretical approach. Ecology. 58, 338–348 (1977).

[b25] Tokeshi M. Species coexistence: Ecological and Evolutionary Perspectives (eds Wiley J. .) Ch. 3, 46–77 (Blackwell Science, 1999).

[b26] Amann R. I., Ludwig W. & Schleifer K. H. Phylogenetic identification and *in situ* detection of individual microbial cells without cultivation. Microbiol. Rev. 59, 143–169 (1995). 7535888 10.1128/mr.59.1.143-169.1995 PMC239358

[b27] McCaig A. E., Grayston S. J., Prosser J. I. & Glover L. A. Impact of cultivation on characterisation of species composition of soil bacterial communities. FEMS Microbiol. Ecol. 35, 37–48 (2001). 11248388 10.1111/j.1574-6941.2001.tb00786.x

[b28] Pham V. H. & Kim J. Cultivation of unculturable soil bacteria. Trends Biotechnol. 30, 475–484 (2012). 22770837 10.1016/j.tibtech.2012.05.007

[b29] Graber J. R. & Breznak J. A. Folate cross-feeding supports symbiotic homoacetogenic spirochetes. Appl. Environ. Microbiol. 71, 1883–1889 (2005). 15812016 10.1128/AEM.71.4.1883-1889.2005 PMC1082566

[b30] Tripp H. J. . Sar11 marine bacteria require exogenous reduced sulphur for growth. Nature. 452, 741–744 (2008). 18337719 10.1038/nature06776

[b31] Stewart E. J. Growing unculturable bacteria. J. Bacteriol. 194, 4151–4160 (2012). 22661685 10.1128/JB.00345-12 PMC3416243

[b32] Eiteman M. A., Lee S. A. & Altman E. A co-fermentation strategy to consume sugar mixtures effectively. J. Biol. Eng. 2, 3 (2008). 18304345 10.1186/1754-1611-2-3 PMC2266900

[b33] Lynd L. R., Weimer P. J., van Zyl W. H. & Pretorius I. S. Microbial cellulose utilization: fundamentals and biotechnology. Microbiol. Mol. Biol. Rev. 66, 506–577 (2002). 12209002 10.1128/MMBR.66.3.506-577.2002 PMC120791

[b34] Szambelan K., Nowak J. & Czarnecki Z. Use of zymomonas mobilis and saccharomyces cerevisiae mixed with kluyveromyces fragilis for improved ethanol production from jerusalem artichoke tubers. Biotechnol Lett. 26, 845–848 (2004). 15269559 10.1023/b:bile.0000025889.25364.4b

[b35] Schwarz W. H. The cellulosome and cellulose degradation by anaerobic bacteria. Appl. Microbiol. Biotechnol. 56, 634–649 (2001). 11601609 10.1007/s002530100710

[b36] Kim T. H., Lee Y. Y., Sunwoo C. & Kim J. S. Pretreatment of corn stover by low-liquid ammonia recycle percolation process. Appl. Biochem. Biotechnol. 133, 41–57 (2006). 16622283 10.1385/abab:133:1:41

[b37] Hui W. . Bioconversion of un-pretreated lignocellulosic materials by a microbial consortium XDC-2. Bioresour. Technol. 136, 481–487 (2013). 23567720 10.1016/j.biortech.2013.03.015

[b38] Lv Z., Yang J., Wang E. & Yuan H. Characterization of extracellular and substrate-bound cellulases from a mesophilic sugarcane bagasse-degrading microbial community. Process Biochemistry. 43, 1467–1472 (2008).

[b39] Wongwilaiwalin S. . Analysis of a thermophilic lignocellulose degrading microbial consortium and multi-species lignocellulolytic enzyme system. Enzyme And Microbial Technology. 47, 283–290 (2010).

[b40] de Lima Brossi M. J., Jiménez D. J., Cortes-Tolalpa L. & van Elsas J. D. Soil-derived microbial consortia enriched with different plant biomass reveal distinct players acting in lignocellulose degradation. Microb. Ecol. 71, 616–627 (2015). 26487437 10.1007/s00248-015-0683-7 PMC4788684

[b41] Takasaki K. . Discovery of glycoside hydrolase enzymes in an avicel-adapted forest soil fungal community by a metatranscriptomic approach. PLOS One. 8, e55485 (2013). 23393585 10.1371/journal.pone.0055485 PMC3564753

[b42] Simmons C. W. . Metatranscriptomic analysis of lignocellulolytic microbial communities involved in high-solids decomposition of rice straw. Biotechnol Biofuels. 7, 495 (2014). 25648696 10.1186/s13068-014-0180-0 PMC4296540

[b43] Jin M., Balan V., Gunawan C. & Dale B. E. Consolidated bioprocessing (CBP) performance of Clostridium phytofermentans on AFEX-treated corn stover for ethanol production. Biotechnol Bioeng. 108, 1290–1297 (2011). 21280028 10.1002/bit.23059

[b44] Moxley G. & Zhang Y. H. P. More Accurate Determination of Acid-Labile Carbohydrates in Lignocellulose by Modified Quantitative Saccharification. Energy & Fuels. 21, 3684–3688 (2007).

[b45] Gilbert J. A., Jansson J. K. & Knight R. The Earth Microbiome project: successes and aspirations. BMC Biol. 12, 69 (2014). 25184604 10.1186/s12915-014-0069-1 PMC4141107

[b46] Gohl D. M. . Systematic improvement of amplicon marker gene methods for increased accuracy in microbiome studies. Nat. Biotechnol. 34, 942–949 (2016). 27454739 10.1038/nbt.3601

[b47] Mitri S., Clarke E. & Foster K. R. Resource limitation drives spatial organization in microbial groups. ISME J. 10, 1471–1482 (2016). 26613343 10.1038/ismej.2015.208 PMC5029182

[b48] Hugenholtz P., Goebel B. M. & Pace N. R. Impact of culture-independent studies on the emerging phylogenetic view of bacterial diversity. J. Bacteriol. 180, 4765–4774 (1998). 9733676 10.1128/jb.180.18.4765-4774.1998 PMC107498

[b49] Ventorino V. . Exploring the microbiota dynamics related to vegetable biomasses degradation and study of lignocellulose-degrading bacteria for industrial biotechnological application. Sci. Rep. 5, 8161 (2015). 25641069 10.1038/srep08161 PMC4648445

[b50] Martins L. F. . Metagenomic analysis of a tropical composting operation at the são paulo zoo park reveals diversity of biomass degradation functions and organisms. PLOS One. 8, e61928 (2013). 23637931 10.1371/journal.pone.0061928 PMC3637033

[b51] Nielsen P. & Sørensen J. Multi-target and medium-independent fungal antagonism by hydrolytic enzymes in Paenibacillus polymyxa and Bacillus pumilus strains from barley rhizosphere. FEMS Microbiology Ecology. 22, 183–192 (1997).

[b52] Mergaert J. . Taxonomic study of Cellvibrio strains and description of Cellvibrio ostraviensis sp. nov., Cellvibrio fibrivorans sp. nov. and Cellvibrio gandavensis sp. nov. Int. J. Syst. Evol. Microbiol. 53, 465–471 (2003). 12710614 10.1099/ijs.0.02316-0

[b53] Hutner S. H., Provasoli L., Schatz A. & Haskins C. P. Some Approaches to the Study of the Role of Metals in the Metabolism of Microorganisms. Proc. Am. Philos. Soc. 94, 152–170 (1950).

[b54] Magoc T. & Salzberg S. L. FLASH: fast length adjustment of short reads to improve genome assemblies. Bioinformatics. 27, 2957–2963 (2011). 21903629 10.1093/bioinformatics/btr507 PMC3198573

[b55] Caporaso J. G. . QIIME allows analysis of high-throughput community sequencing data. Nature Methods. 7, 335–336 (2010). 20383131 10.1038/nmeth.f.303 PMC3156573

[b56] Edgar R. C. Search and clustering orders of magnitude faster than BLAST. Bioinformatics. 26, 2460–2461 (2010). 20709691 10.1093/bioinformatics/btq461

[b57] Edgar R. C., Haas B. J., Clemente J. C. Quince C. & Knight R. UCHIME improves sensitivity and speed of chimera detection. Bioinformatics 27, 2194–2200 (2011). 21700674 10.1093/bioinformatics/btr381 PMC3150044

[b58] Desantis T. Z. . Greengenes, a chimera-checked 16S rRNA gene database and workbench compatible with ARB. Appl. Environ. Microbiol. 72, 5069–5072 (2006). 16820507 10.1128/AEM.03006-05 PMC1489311

[b59] Hammer O., Harper D. A. T. & Ryan P. D. PAST: paleontological statistics software package for education and data analysis. Palaeontologia electronica. 4 (2001).

