# Supplementary material for: Nutrient availability shapes the microbial community structure in sugarcane bagasse compost-derived consortia
Source: Sci Rep. 2016 Dec 12;6:38781. doi: 10.1038/srep38781 (PMC5150498; doi:10.1038/srep38781)
Supplement: Supplementary Information [file srep38781-s1.pdf]

Supplementary Information

Nutrient availability shapes the microbial community structure in sugarcane bagasse compost-derived consortia

Bruno L. Mello[1]*, Anna M. Alessi[2]*, Simon McQueen-Mason[2], Neil C. Bruce[2], and Igor Polikarpov[1]

* These authors contributed equally to this work.

[1]Instituto de Física de São Carlos, Universidade de São Paulo, Av. Trabalhador São-carlense 400, São Carlos, SP, 13560-970, Brazil, e-mails: bruno.luan.mello@usp.br; ipolikarpov@ifsc.usp.br

[2]Department of Biology, University of York, Wentworth Way, York, UK, YO10 5DD, emails: anna.szczepanska@york.ac.uk; simon.mcqueenmason@york.ac.uk; neil.bruce@york.ac.uk

Corresponding author Igor Polikarpov, Instituto de Física de São Carlos, Universidade de São Paulo, Av. Trabalhador São-carlense 400, São Carlos, SP, 13560-970, Brazil

Phone +55 16 3373-8088, fax +55 16 3373-9881, email: ipolikapov@ifsc.usp.br

Supplementary Figure 1

Qualitative overview of sugarcane bagasse biomass collected from MM and PCS grown compost-derived consortia during five-week time course showing morphological changes such as particle size reduction, color darkening and biomass softening.

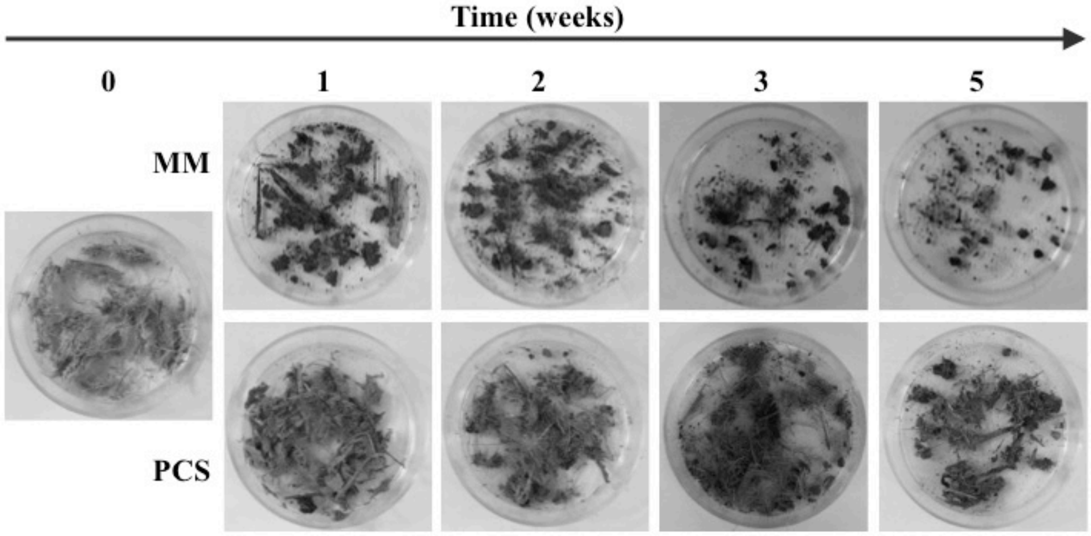

Supplementary Table 1

Relative abundance values for twenty most abundant OTUs present in a) MM- and b) PCS-grown communities. Bolded taxa are present in both tables.

A)

phylum	class	order	family	genus	Mean abundance in MM community (%)	Mean abundance in PCS community (%)	Cumulative contribution to dissimilarity in MM (%)
Actinobacteria	Actinobacteria	Actinomycetales	Cellulomonadaceae	Cellulomonas	6.7	0.0	6.7
Acidobacteria	**Chloracidobacteria**	**RB41**	**Ellin6075**	**unknown**	5.8	1.3	12.5
Actinobacteria	Actinobacteria	Actinomycetales	unknown	unknown	4.8	0.5	17.3
Bacteroidetes	Flavobacteriia	Flavobacteriales	Cryomorphaceae	unknown	3.6	0.0	21.0
Firmicutes	Bacilli	Bacillales	Paenibacillaceae	Paenibacillus	3.2	0.1	24.1
Proteobacteria	γ-proteobacteria	Alteromonadales	Alteromonadaceae	unknown	3.0	0.3	27.1
Bacteroidetes	**Saprospirae**	**Saprospirales**	**Chitinophagaceae**	**unknown**	2.6	5.5	29.7
Proteobacteria	γ-proteobacteria	Xanthomonadales	Xanthomonadaceae	unknown	2.3	0.1	32.0
Proteobacteria	**α-proteobacteria**	**Caulobacterales**	**Caulobacteraceae**	**Asticcacaulis**	2.3	7.6	34.3
Proteobacteria	α-proteobacteria	Caulobacterales	Caulobacteraceae	unknown	2.3	0.8	36.6
Proteobacteria	α-proteobacteria	Rhizobiales	unknown	unknown	2.2	0.9	38.8
Proteobacteria	γ-proteobacteria	Xanthomonadales	Sinobacteraceae	unknown	2.1	0.3	40.9
other kingdom	-	-	-	-	2.0	1.8	43.0
Planctomycetes	Planctomycetia	Pirellulales	Pirellulaceae	unknown	2.0	0.6	45.0
Planctomycetes	Planctomycetia	Pirellulales	Pirellulaceae	A17	1.9	0.0	46.8
Chloroflexi	Anaerolineae	Caldilineales	Caldilineaceae	unknown	1.8	0.9	48.7
Proteobacteria	**α-proteobacteria**	**Rhizobiales**	**Hyphomicrobiaceae**	**Devosia**	1.7	3.0	50.4
Bacteroidetes	**unknown**	**unknown**	**unknown**	**unknown**	1.7	2.0	52.1
Planctomycetes	Planctomycetia	Planctomycetales	Planctomycetaceae	Planctomyces	1.7	0.6	53.8
Proteobacteria	γ-proteobacteria	Xanthomonadales	Xanthomonadaceae	unknown	1.6	0.5	55.4
Proteobacteria	α-proteobacteria	Rhodobacterales	Hyphomonadaceae	unknown	1.6	0.0	57.0

B)

phylum	class	order	family	genus	Mean abundance in MM community (%)	Mean abundance in PCS community (%)	Cumulative contribution to dissimilarity in PCS (%)
Bacteroidetes	Saprospirae	Saprospirales	Chitinophagaceae	unknown	0.9	10.2	10.2
Bacteroidetes	Saprospirae	Saprospirales	Saprospiraceae	unknown	0.6	10.0	20.2
Proteobacteria	**α-proteobacteria**	**Caulobacterales**	**Caulobacteraceae**	**Asticcacaulis**	2.3	7.6	27.9
Bacteroidetes	Cytophagia	Cytophagales	unknown	unknown	1.5	6.2	34.1
Bacteroidetes	**Saprospirae**	**Saprospirales**	**Chitinophagaceae**	**unknown**	2.6	5.5	39.6
Bacteroidetes	Sphingobacteriia	Sphingobacteriales	Sphingobacteriaceae	unknown	0.1	4.5	44.1
Bacteroidetes	Sphingobacteriia	Sphingobacteriales	Sphingobacteriaceae	Sphingobacterium	0.0	3.6	47.7
Proteobacteria	**α-proteobacteria**	**Rhizobiales**	**Hyphomicrobiaceae**	**Devosia**	1.7	3.0	50.7
Bacteroidetes	Flavobacteriia	Flavobacteriales	Weeksellaceae	unknown	0.0	3.0	53.6
Chloroflexi	Anaerolineae	SBR1031	A4b	unknown	0.7	2.7	56.3
Proteobacteria	γ-proteobacteria	Alteromonadales	Alteromonadaceae	Cellvibrio	1.5	2.6	58.9
Bacteroidetes	Cytophagia	Cytophagales	Cytophagaceae	Leadbetterella	0.9	2.3	61.2
Bacteroidetes	**unknown**	**unknown**	**unknown**	**unknown**	1.7	2.0	63.2
other kingdom	-	-	-	-	2.0	1.8	65.0
Proteobacteria	α-proteobacteria	BD7-3	unknown	unknown	0.1	1.7	66.8
Firmicutes	Bacilli	Bacillales	Planococcaceae	unknown	0.0	1.7	68.4
Verrucomicrobia	Spartobacteria	Chthoniobacterales	Chthoniobacteraceae	heteroC45_4W	0.1	1.5	70.0
Bacteroidetes	Flavobacteriia	Flavobacteriales	Flavobacteriaceae	unknown	0.4	1.5	71.5
Acidobacteria	**Chloracidobacteria**	**RB41**	**Ellin6075**	**unknown**	5.8	1.3	72.8
Proteobacteria	α-proteobacteria	Rhodobacterales	Rhodobacteraceae	unknown	1.2	1.2	74.0
Proteobacteria	α-proteobacteria	Rhizobiales	Hyphomicrobiaceae	unknown	0.9	1.1	75.1
